# Ricin: an ancient toxicant, but still an evergreen

**DOI:** 10.1007/s00204-023-03472-w

**Published:** 2023-03-07

**Authors:** Hermann M. Bolt, Jan G. Hengstler

**Affiliations:** Leibniz Research Centre for Working Environment and Human Factor at TU Dortmund (IfADo), Ardeystr. 67, 44139 Dortmund, Germany

Reports in the lay press on intended terrorist attacks with ricin or castor seeds have reached recent publicity (Anonymous 2023; Noryskiewicz 2023), reminiscent of earlier cases in the United States (Audi et al 2005) and elsewhere (Abbes et al 2021). The most famous classical case is that of Georgi Markov, working for BBC World Service, who had been killed in London, on 11 Sep 1978, by a poison dart from an umbrella, which was filled with a tiny pellet of ricin (”*umbrella murder*”). After this incident, Georgi Markov developed high temperature and died within 4 days (Crompton and Gall 1980, Nepovimoca and Kuca 2019). Cases of intoxication by castor seeds were also published in the past by Archives of Toxicology: two fatalities in Budapest/Hungary (Balázs 1933), one fatality in Turkey (Abdülkadir-Lütfi and Taeger 1935), and ten cases of school children that survived the intoxication (Karszás and Papp 1960).

Castor seeds have been known as drug and toxicant since ancient times and were found in Egyptian tombs dating 4000 BC (Poelchen and Wirkner 2003). Ricin, the toxic principle of castor seeds (*Ricinus communis L.*) was first described in 1888 by Stillmark at the University of Dorpat (now Tartu, Estonia), an important centre of pharmacology in the late nineteenth century (Boehm 1874). Owing to its easy availability and high toxicity, *Ricinus communis L.* was awarded the dubious title “poisonous plant of the year 2018” in Germany (Franke et al 2019). The toxicity of ricin is estimated to be approximately 380-fold higher than that of the nerve agent VX (Liang et al 2021). A number of recent reviews cover different aspects of ricin, its preparation from castor seeds and its potential use as a biological weapon (Griffiths 2011; Olsnes and Kozlov 2001; Poelchen and Wirkner 2003; Spivak and Hendrickson 2005; Polito et al 2019). Owing to its ease of preparation, wide availability, and potential use as a bioterrorism agent, ricin is listed as a prohibited substance under schedule 1A of the Chemical Weapons Convention (CWC, Liang et al 2021). Ricin research is now focused on the following aspects.

## Mode of toxic action

In general, type-II ribosome-inactivating proteins (RIPs) are an important class of protein toxins that consist of A and B chains linked by an inter-chain disulfide bond. It has been recognized since long that isolated A and B chains are not toxic (Creppy et al 1980). More recent research has shown that the B-chain with lectin-like activity is responsible for binding to the galactose-containing receptors on eukaryotic cell surfaces, which is essential for internalization of the A-chain by endocytosis. Translocation of the A-chain is preceded by endocytic uptake and retrograde traffic through the trans-Golgi network and the endoplasmic reticulum (Sowa-Rogozinska et al 2019). The A-chain has *N*-glycosidase activity that leads to irreversible depurination of adenine from 28S ribosomal RNA and thus terminates protein synthesis. This synergism of the A and B chains inactivates the ribosome and leads to high cytotoxicity after oral administration or inhalation (Liang et al 2021).

## Analytical detection

Techniques for the detection of ricin A have been reviewed in detail (Bozza et al 2015). There is a continuous development in this field. Basically, assays distinguishing between biologically active and inactive ricin are essential for evaluating both the lethality of a bioterrorism threat and the monitoring of decontamination procedures. Biological assays have limitations in selectivity and cannot distinguish ricin from related toxins. An integrated approach should, therefore, be used. An optimal assay design includes a rapid and efficient enrichment step, an A-chain activity checkpoint, and a selectivity step distinguishing ricin from other bioactive toxins. Assays have been developed, where ricin is enriched from samples using ricin-specific antibodies. However, this procedure is unable to confirm B-chain lectin functionality and, therefore, does not guarantee that the sequestered ricin is capable of penetrating a host cell membrane and exert toxicity. A viable approach for sample enrichment would be the use of sugar-conjugated materials that exploit the lectin-binding properties of the ricin B-chain. Several glycosphingolipids and synthetic sugars bind to ricin with high affinity. After sample enrichment, mass spectrometry may simultaneously identify ricin peptide fingerprints and in vitro RNA substrates that have been deadenylated by the ricin A-chain and would allow for quick and sensitive detection of biological active ricin. This assay could detect functional B-chain necessary for cell penetration, confirm A-chain activity required to inactivate the ribosome, and selectively identify ricin as the harmful toxin. Additionally, a robust cell-based assay for ricin cytotoxicity can be used as a confirmatory test (Bozza et al 2015).

## New avenues for therapy

Currently no antidote, vaccine, or other specific effective treatment is available for ricin poisoning or prevention. Treatment with supportive care is still the only means to limit morbidity and mortality (Abbes et al 2021). Over the past 20 years, research has been performed to treat or prevent ricin poisoning with neutralizing antibodies (for review, see Yu et al 2022). In principle, neutralizing antibodies may effectively counteract ricin toxicity. But most anti-ricin antibodies have shown no neutralizing activity or showed neutralizing activity only in vitro. Antibodies directed against epitopes close to the binding interface of the A and B chain of ricin have a better neutralization performance (Rudolph et al 2021). According to recent evidence antibody cocktails consisting of chain A and chain B antibodies show better neutralizing activity at lower antibody doses compared to antibodies directed against only one chain (Rong et al 2020, Orsini Delgado 2021). Such promising experimental data now call for in-depth clinical studies (Yu et al 2022).

Thus, although being known as drug and toxicant since ancient times, ricin is a matter of active and highly interesting contemporary research.
